# How does a short period of exercise effect toe pressures and toe-brachial indices? A cross-sectional exploratory study

**DOI:** 10.1186/s13047-018-0309-7

**Published:** 2018-11-26

**Authors:** Peta Ellen Tehan, Sean George Sadler, Sean Michael Lanting, Vivienne Helaine Chuter

**Affiliations:** 1 School of Health Sciences, Faculty of Health and Medicine, University of Newcastle, Ourimbah, NSW 2258 Australia; 2 Priority Research Centre for Physical Activity and Nutrition, Univeristy of Newcastle, Callaghan, 2308 NSW Australia

**Keywords:** Post exercise TBI, Post exercise toe pressure, Toe pressure, Toe-brachial pressure, Exercise, Peripheral arterial disease, Lower extremity

## Abstract

**Background:**

Whilst post exercise ankle-brachial indices (ABI) are commonly used to help identify peripheral arterial disease (PAD), the role of post exercise toe pressures (TP) or toe-brachial indices (TBI) is unclear**.** The aim of this study was to determine, in a population without clinical signs of PAD, the effect that 30 s of weight-bearing heel raises has on TP and TBI values. Additionally, the ability of resting TP and TBI values to predict change in post-exercise values using the heel raise method was investigated.

**Methods:**

Participants over the age of 18 with a resting TBI of ≥0.60 and ABI between 0.90 and 1.40, without diabetes, history of cardiovascular disease and not currently smoking were included. Following ten minutes of supine rest, right TP and bilateral brachial pressures were performed in a randomized order using automated devices. Participants then performed 30 s of weight-bearing heel raises, immediately after which supine vascular measures were repeated. Data were assessed for normality using the Shapiro-Wilk test. For change in TP and TBI values the Wilcoxon Signed-Rank Test was performed. For correlations between resting and change in post exercise values, the Spearman Rank Order Correlations were performed, and where significant correlation identified, a linear regression undertaken.

**Results:**

Forty-eight participants were included. A statistically significant decrease was seen in the median TP from resting 103.00 mmHg (IQR: 89.00 to 124.75) to post exercise 98.50 mmHg (IQR: 82.00 to 119.50), z = − 2.03, *p* = 0.04. This difference of 4.50 mmHg represents a 4.37% change and is considered a small effect size (*r* = 0.21). The median TBI also demonstrated a statistically significant decrease from resting 0.79 (IQR: 0.68 to 0.94) to post exercise 0.72 (IQR: 0.60 to 0.87), z = − 2.86, *p* = < 0.01. This difference of 0.07 represents an 8.86% change and is considered a small effect size (*r* = 0.29). Linear regression demonstrated that resting TBI predicted 22.4% of the variance in post exercise TBI, *p* = < 0.01, coefficients beta − 0.49.

**Conclusions:**

Thirty seconds of weight-bearing heel raises resulted in a similar decrease in TBI values seen in longer periods of exercise. TP values also showed a decrease post exercise; however this was contrary to previous studies.

## Introduction

Podiatrists complete an average of two non-invasive lower limb vascular assessments per day [1], making it a key part of clinical practice, and placing them at the forefront of detection of peripheral arterial disease (PAD). Commonly used vascular assessment techniques by podiatrists include ankle-brachial indices (ABI) and toe-brachial indices (TBI) [1], which are ideally performed after a period of ten minutes of horizontal supine rest [2]. Both tests assess the ratio of peripheral systolic pressures (ankle and toe respectively) to brachial pressure. For the ABI, a post exercise measurement can also be used. In this instance, pressures are taken directly after a brief period of exercise and compared to the resting ABI. This additional testing is particularly useful in patients where PAD is suspected, however the resting ABI is normal. A decrease in the post exercise ABI value of 20% or more compared to the resting ABI is indicative of clinically significant PAD. In a healthy population, the post exercise ABI should be similar to or slightly lower than the resting ABI, due to a decrease in vascular resistance [3, 4].

Previous research has demonstrated that the post exercise ABI has superior sensitivity for detecting PAD compared to a resting ABI in those with suspected or symptomatic PAD, or where PAD diagnosis is uncertain [4, 5]. In populations where the ABI has been shown to have poor diagnostic accuracy for PAD, for example those with renal disease, those of advanced age and in diabetes cohorts, a resting TBI is frequently used as an alternative index test [6, 7]. There is a growing amount of research supporting the use of the TBI in such populations with evidence of superior diagnostic accuracy to the ABI [6, 8, 9]. However, little research has investigated the potential for the diagnostic capacity of this test to be extended by using it post exercise in the same manner as the post exercise ABI.

Research investigating the effect of exercise on the TBI is limited. A single study has investigated the effect of five minutes of graded treadmill exercise or a six minute walk test on TP and TBI measures [10] in 120 people with PAD and a control population (*N* = 30). Using laser-Doppler fluxmetry to measure TP, the study demonstrated in the diseased population a significant reduction in TP and TBI measures post exercise. The post exercise measures also had a greater capacity to identify critical limb threatening ischaemia than resting measures. Conversely, in the control population there was a small increase in TP (+ 4 mmHg) post exercise, whereas TBI (− 0.05) showed an overall decrease. While these findings suggest that post exercise TP and TBI measures may be useful for improving the diagnostic accuracy of clinical assessment for PAD, use of this test will be limited by both clinical facilities to conduct the testing and patient capacity to undertake the exercise. For the post exercise ABI it has been demonstrated that 30 s of weight-bearing heel raises correlates with changes in ABI values seen after longer periods of treadmill exercise [11]. This provides an alternative option for performing exercise testing in a clinical environment where graded treadmill exercise may not be possible. However, it is unknown if 30 s of heel raises causes similar changes to those demonstrated with treadmill exercise for post exercise TP or TBI.

Therefore, the primary aim of this exploratory, cross-sectional study was to determine, in a population with a resting TBI ≥0.60 and ABI of 0.90 to 1.40, the effect that 30 s of weight-bearing heel raises has on TP and TBI values. Additionally, the ability of resting TP and TBI values to predict change in post exercise values using the heel raise method was investigated.

## Methods

This study was undertaken at the University of Newcastle Podiatry teaching clinics located at the Newcastle Community Health Centre and Wyong Hospital, NSW, Australia. Ethics approval was obtained from the University of Newcastle Human Research Ethics Committee (H-2016-0340). Participants were recruited on a volunteer basis from patients and students attending both clinics. All participants gave written informed consent prior to participation. Participants were included if they were over the age of 18, were able to perform 30 s of heel raises and able to have systolic ankle, toe and brachial pressures measured. Additionally, participants were only included if they had a resting TBI of ≥0.60 and resting ABI between 0.90 to 1.40 [12, 13]. Participants were excluded if they were currently smoking or if they had: loss of neurological sensation, symptoms of intermittent claudication, a history of myocardial infarction or cerebrovascular event, a recent ankle sprain or other lower leg injury that may have prevented neurological or vascular assessment, known allergy to coupling gel, bilateral mastectomy precluding brachial pressure measurement, lower limb lymphoedema, right hallux amputation, an active lower limb wound, Raynaud’s disease, scleroderma or other vasospastic disorder known to interfere with accuracy of vascular measurements.

Participants attended the clinic for a single testing session and were asked to avoid caffeine and exercise for one hour prior to the testing session as this is known to affect blood pressure measurement [14]. Demographic data, medical history, smoking status, and history of cardiovascular disease or vascular related lower limb complications were obtained from all participants by self-report. Participants then underwent lower limb neurological and vascular testing. All measurements were taken by one of two testers (SL, SS), both practicing podiatrists with 6 years of clinical experience. To reduce the incidence of type 1 error, all lower limb measurements were undertaken on the right limb only [15].

### Testing procedure

Neurological testing was undertaken prior to lower limb vascular assessments and conducted with the patient in supine lying position. The neurological assessment consisted of a four-point monofilament test [16]. A fail for the four-point monofilament test occurred if one or more of the sites were missed and participants were excluded from participation in the study due to the potential for abnormal sensation.

Prior to lower limb vascular assessment participants were rested for 10 min in a horizontal supine position in a quiet, temperature controlled (23–25 degrees Celsius) clinical room [17]. Bilateral brachial blood pressures were measured using a MicroLife BP A100 Plus (MicroLife AG, Widnau, Switzerland) automated unit [3]. The MicroLife BP A100 Plus device has been shown to be valid and reliable [18]. TP was measured at the hallux using a Systoe® (Atys Medical, Soucieu-en-Jarrest, France) automated device which has previously been shown to have excellent reliability [19]. Systolic ankle blood pressures were measured using a Doppler and a manual sphygmomanometer. Consistent with international guidelines, the ABI was calculated by dividing the highest of the two ankle pressures (dorsalis pedis or posterior tibial) by the highest of the bilateral brachial pressures [3]. Similarly, the TBI was calculated by dividing the TP by the highest of the bilateral brachial pressures [3]. To reduce the chance of an order effect, the order of the vascular testing for each participant was randomized using Microsoft Excel.

After resting vascular assessments were conducted, participants performed weight-bearing heel raises for a total of 30 s to the participant’s maximum achievable height, and at a recommended rate of one heel raise per second [11]. TP and brachial pressures were then re-measured immediately after completion of the heel raises. The post exercise blood pressures were measured in the same order with the participant in the same position as they were prior to exercise. Due to the ABI measurement including manual ankle pressures, inter-tester reliability was established in the two testers (SL, SS) by completing ankle pressure measurements in a separate group of healthy volunteers (*N* = 27) within a single testing session. The same testing protocol was followed as listed above. Testers were blinded to each other’s results, and the participant was given a ten minute break between testing. All other vascular measures were completely automated and have established acceptable levels of reliability [19].

### Statistical methods

Microsoft Excel was used for data entry and preparation with SPSS version 24 used for statistical analysis. Data were assessed for normality using descriptive statistics such as histograms, boxplots, and the Shapiro-Wilk test. For non-normally distributed data, medians and interquartile ranges were calculated where required.

Inter-tester reliability of ankle pressures was determined using intra-class correlation coefficients (ICC, 2, 1) with 95% confidence intervals (95%CI). Dorsalis pedis or posterior tibial artery pressures of the same leg were randomly selected for each participant and included in the analysis. All ICC values for inter-tester reliability were interpreted according to cut-offs suggested by Fleiss [20].To compare resting and post exercise TP and TBI values, due to the presence of non-parametric data, a Wilcoxon Signed-Ranked Test was performed. Effect sizes were calculated using Microsoft Excel and reported using the z value statistic divided by the square root of N (cases × 2) with the size of the effect interpreted according to Cohen [21]: 0.1–0.29 = small effect; 0.3–0.49 = medium effect; ≥0.5 = large effect.

For correlations, Spearman Rank Order Correlation was performed as the intended dependent variables (TP and TBI change) were non-normally distributed. Resting TP, TBI, and brachial values were the independent variables for each correlation matrix. Assumption of related pairs, independence of observations, normality, linearity, and homoscedasticity were assessed using the Shapiro-Wilk test, as well as scatterplots.

A linear regression analysis was performed due to the resting TBI being significantly negatively correlated with change in post exercise TBI values (*p* < 0.05). No other significant correlations were identified for change in TP and TBI values following exercise. Assumptions of multicollinearity, linear relationship between the independent and dependent variable, the regression being multivariate normal, no auto-correlation, and homoscedasticity were adhered too, as assessed in the SPSS output. No outlier data were found in the analyses.

## Results

**Fig. 1:**
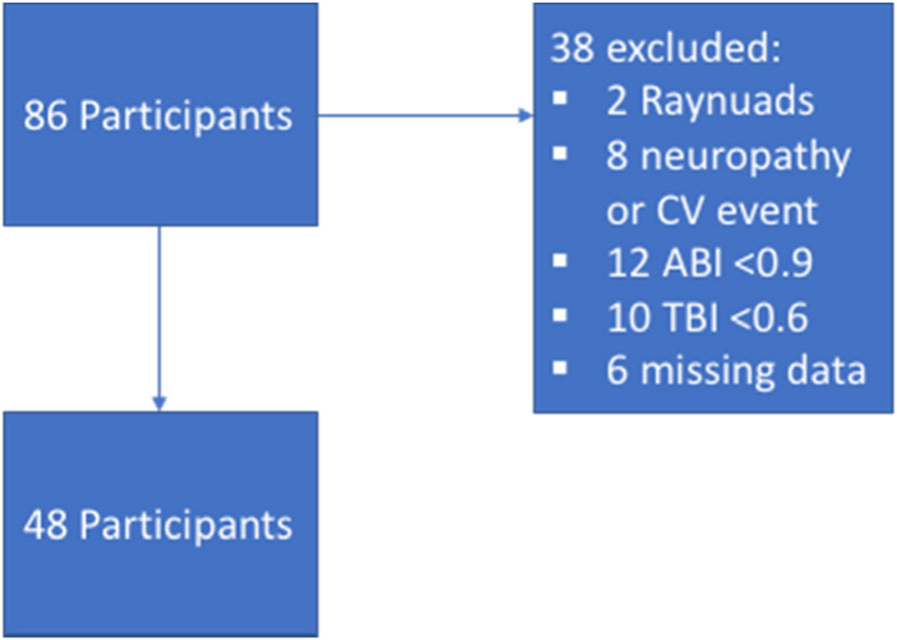
Flowchart for participant inclusion/exclusion

Eighty-six participants were recruited however after applying screening and exclusion criteria, forty-eight participants were included in the final analysis (Fig. 1), with participant characteristics outlined in Table 1. Median age of participants was 27 years (IQR: 22 to 62) and twenty-six (54%) were female. Inter-tester reliability for ankle pressures was good ICC (2, 1): 0.77 (95% CI 0.55 to 0.88, *p* < 0.0001).

**Table 1:** Participant Characteristics updated

Participant Characteristics	
N	48
Female n (%)	26 (54)
Age range (years)	19–90
Median age (years, IQR)	27 (39.5)

**Table 2:** Pre-Exercise and Post exercise TP, TBI and BP values

Measurement	N	Pre-Exercise	Post exercise	Pressure change (% of resting)	Z	p	Effect size
Median TP mmHg (IQR)	48	103.00 (89.00 to 124.75)	98.50 (82.00 to 119.50)	−4.5 (4.37)	−2.03	0.04	*r* = 0.21 (small effect)
Median TBI (IQR)	48	0.79 (0.68 to 0.94)	0.72 (0.60 to 0.87)	−0.07 (8.86)	−2.86	0.004	*r* = 0.29 (small effect)
Median BP mmHg (IQR)	48	128 (120.25 to 138.50)	133 (124.25 to 144.50)	+ 5.0 (3.90)	−3.23	0.001	*r* = 0.33 (medium effect)

*TP* toe systolic pressure, *IQR* inter-quartile range, *TBI* toe-brachial index, *BP* blood systolic pressure

**Fig. 2:**
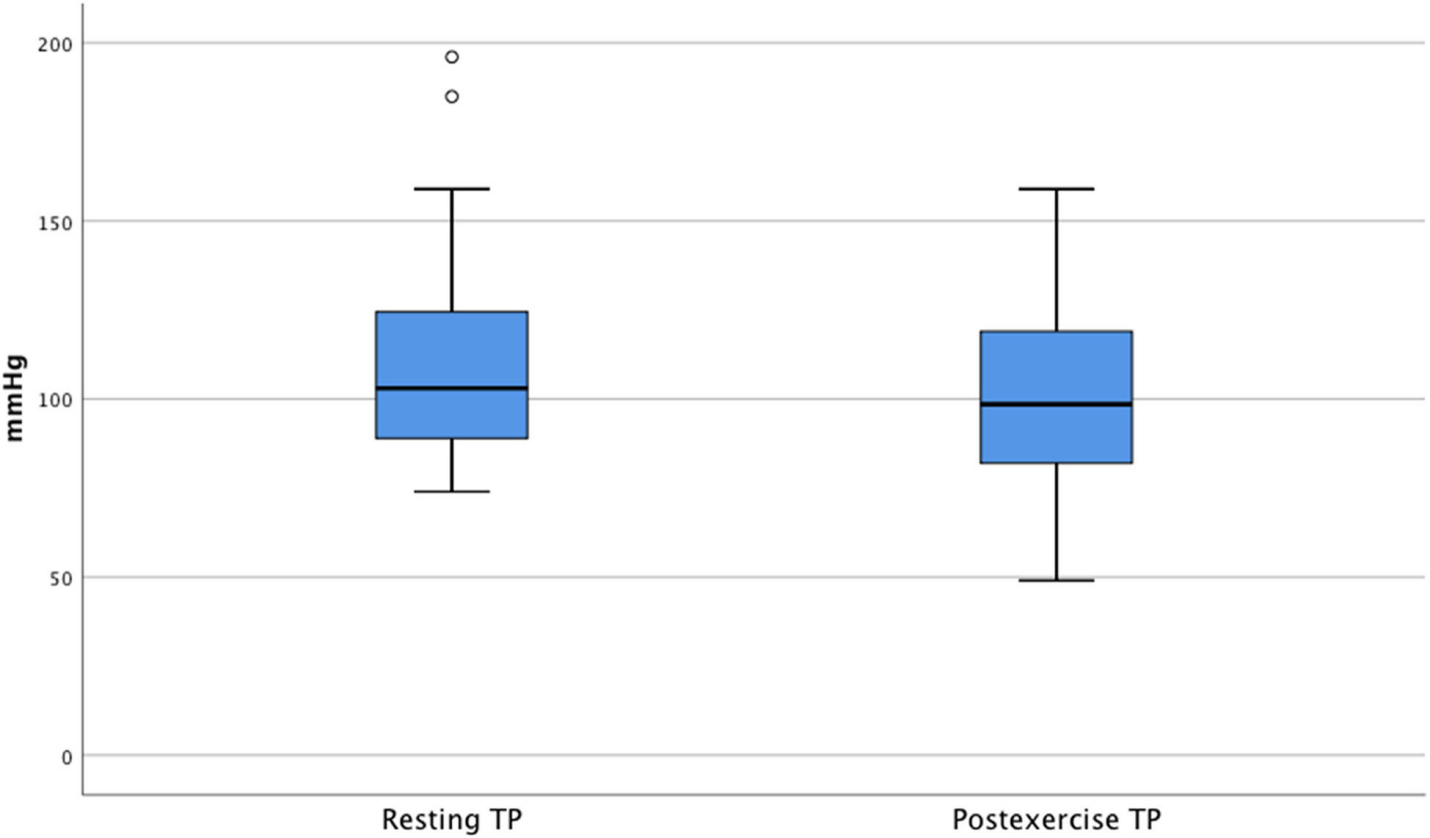
Boxplot of resting and postexercise systolic toe pressure (TP)

**Fig. 3:**
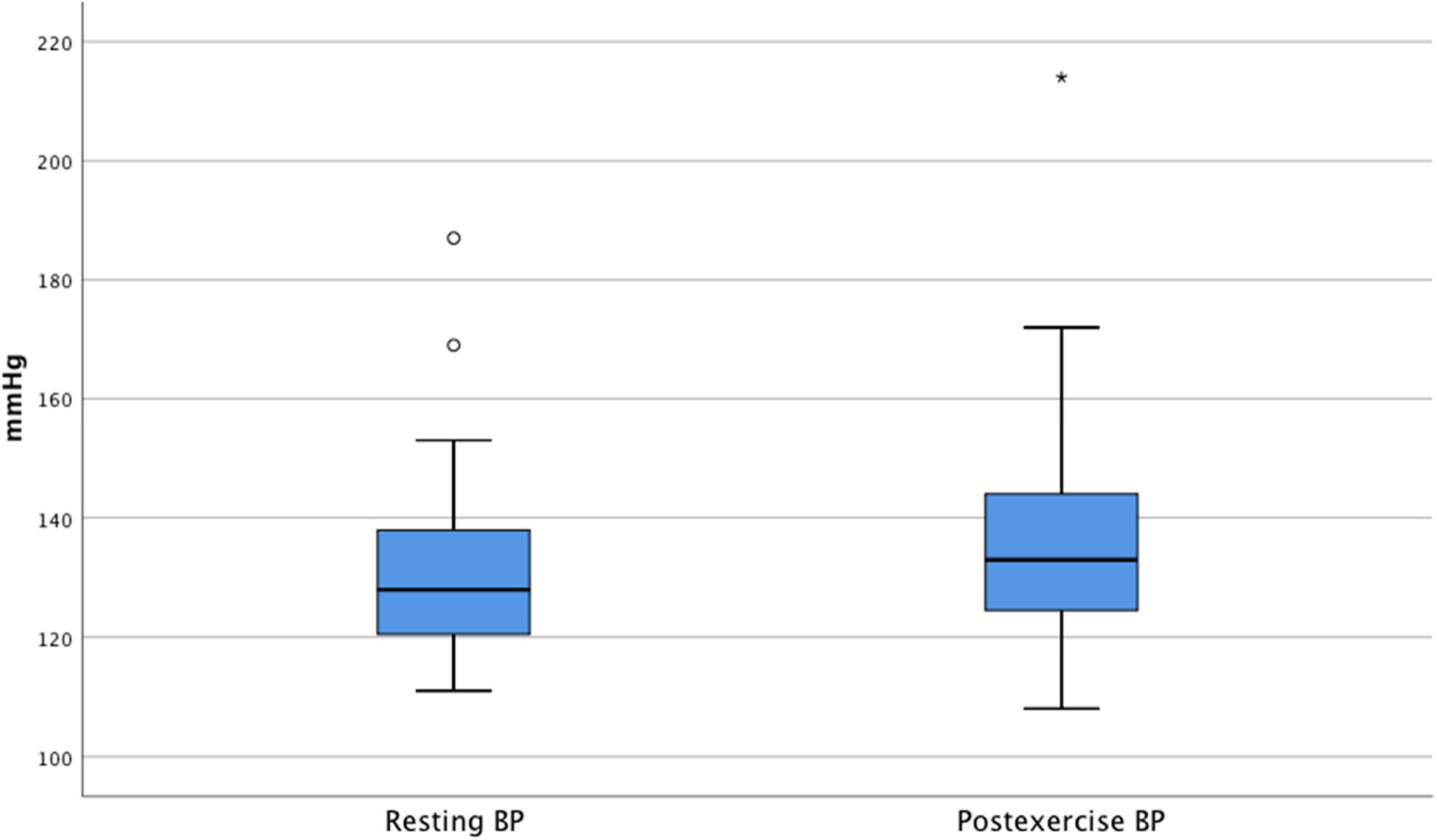
Boxplot of resting and postexercise systolic brachial pressure (BP)

**Fig. 4:**
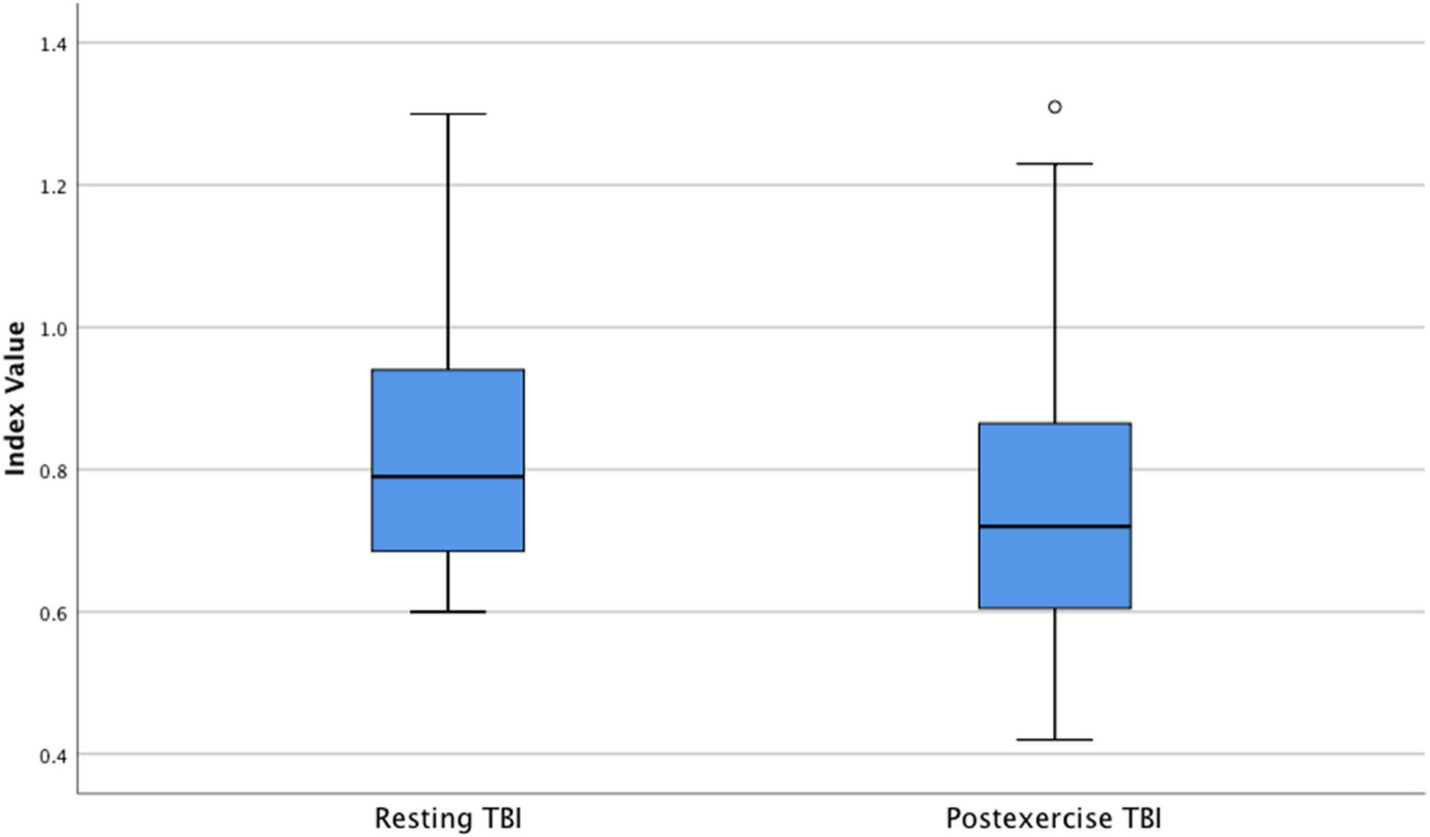
Boxplot of resting and postexercise toe-brachial index (TBI)

TP demonstrated a statistically significant reduction from resting to post exercise with median drop of 4.50 mmHg (Table 2). The median TP value decreased from resting 103.00 mmHg (IQR: 89.00 to 124.75) to 98.50 mmHg (IQR: 82.00 to 119.50 mmHg) post exercise (z = − 2.03, *p* = 0.04), with a small effect size (*r* = 0.21) (Fig. 2). Median systolic brachial pressure demonstrated a significant increase in the median post exercise pressure of 5.00 mmHg, rising from 128.00 mmHg (IQR: 120.25 to 138.50) resting to 133.00 mmHg (IQR: 124.25 to 144.50 mmHg) post exercise (z = − 3.23, *p* = < 0.01), with a medium effect size (*r* = 0.33) (Fig. 3). The median TBI had a statistically significant decrease of 0.07, decreasing from 0.79 (IQR: 0.68 to 0.94) at rest to 0.72 (IQR: 0.60 to 0.87) post exercise (*z* = − 2.86, *p* = < 0.01) with a small effect size (*r* = 0.29) (Fig. 4).

Linear regression demonstrated that resting TBI predicted approximately 22.4% of the variance seen in change in post exercise TBI (adjusted r^2^ 0.22, *p* = < 0.01, coefficients beta − 0.49).

## Discussion

The current study aimed to determine the effect of 30 s of weight-bearing heel raise exercises on TP and TBI compared to resting measures in a population with a resting TBI ≥0.60 and ABI 0.90 to 1.40. Secondly, this study aimed to investigate the ability of resting TP and TBI values to predict change in post exercise values of these measures.

The results of this study showed that in our participant population, TP demonstrated an overall reduction following 30 s of heel raises. For TP the median value of the post exercise TP was 4.50 mmHg lower than the resting TP median value. Conversely, the median value for brachial pressures was higher post exercise increasing by 5.00 mmHg. Nevertheless, the TBI dropped post exercise, with the median value reducing by 0.07 which represents an 8.86% decrease. All these changes were statistically significant. Recent research investigating the effect of 5 min of treadmill walking, or a 6 min walk test, in people with diagnosed PAD and a healthy control group, demonstrated increases in both ankle and toe pressures in those without PAD but an overall reduction in both ABI and TBI (mean drop 0.05 for both) ratios [10]. The current study demonstrated a reduction in TP post exercise, contrary to Kovacs et al. (2018) who demonstrated an increase in TP post exercise. Whilst these directions of change were contrary, they were of a similar magnitude. The TBI however, dropped in a similar fashion to the previous study (0.05 vs 0.07). The difference in directional change in TP between our present study and Kovacs et al. (2018) may have been associated with methodological differences between the studies (e.g. laser Doppler versus photoplethysmography for TP measurements) [10]. However, given the relatively small magnitude of change in both studies, it is unlikely to be of clinical significance, particularly given the relatively wide 95% limits of agreement reported for TP in numerous studies previously [22, 23]. Essentially, the two studies both determined that in a population apparently without PAD, the TP will not demonstrate a very large change post exercise and does not result in abnormal TP values.

Overall, the consistency of the TBI response to exercise between this study and a previous study, which exercised participants with up to 5 min of treadmill walking or a 6-min walk test, suggests that 30 s of heel raises may be an alternative method of performing a post exercise TBI. Given that podiatrists have reported their main barrier for completing vascular assessments is a lack of time [1], this makes the results of this study clinically relevant, as we were able to replicate the results of a longer testing protocol in a shorter timeframe. Furthermore, the current testing protocol is not overly strenuous, so may be more suitable for patients with limited mobility or those patients who are unable to walk on a treadmill or walk for lengthier periods.

This present study showed that the resting TBI was significantly, moderately, negatively correlated with changes in post exercise TBI values. This suggests that higher resting TBI values resulted in a greater index drop post exercise, compared to lower resting TBI values. Due to the lack of research investigating the use of post exercise TP and TBI, there are no published parameters for interpretation of the magnitude of changes in the measurement between resting and post exercise testing. Nevertheless it is likely that, consistent with the current assumption on the post exercise ABI [12], that the reduction in peripheral pressure induced by exercise is a result of a decrease in peripheral vascular resistance. Given that our study included people with ABI between 0.90 and 1.40 and TBI values ≥0.60, these data cannot be extrapolated to populations with significant PAD. It is also an assumption that these clinical tests excluded those with significant PAD. High body mass index and sedentary lifestyle are risk factors for PAD, and post exercise TP and TBI should be investigated in these populations. In addition, the capacity to perform the exercises in some populations may influence the tests diagnostic accuracy. Further research needs to be undertaken evaluating post exercise TP and TBI in participants not only with and without PAD but also with varying extents of PAD using imaging as the reference standard. This will assist with determining the capacity of the post exercise TBI to help determine disease severity.

Regression analysis determined that the resting TBI predicted 22.40% of the variance in post exercise TBI values. The remainder of the variance seen in post exercise TBI measurement in our present study may be due to a number of different factors which were not measured in this study. Participants’ level of regular physical activity may influence microvascular reactivity. Multiple studies have identified both enhanced endothelial-dependent cutaneous vasodilation [24] and increased resting skin blood flow [25–28] in highly trained athletes and those with higher levels of self-reported physical activity, respectively, compared to less physically active controls. Measurement error may also be a factor influencing post exercise, due to the very small changes seen in pressure post exercise, it may be difficult to accurately quantify a change of that magnitude. Furthermore, although every effort was made to control the influence of environmental factors and exclude those with preexisting conditions known to influence TP, it is still possible these may have influenced the test outcomes. Of particular note, we were not able to confidently exclude the presence of PAD in the absence of diagnostic imaging, however, used a number of subjective and objective clinical assessment tools (claudication history, ABI and TBI) to exclude participants with any abnormal findings which may have suggested the presence of PAD, so the inclusion of participants with PAD was unlikely.

### Potential limitations

The findings of this study should be considered in light of some further limitations. There are a number of variables that were not measured in this study that may have influenced TP and TBI values. Skin temperature was not measured, and it may have changed over the 10 min resting period however the room was temperature controlled so this is unlikely. This study used heel raises as an exercise method, which has previously been validated in a claudicant population. It is possible that a similar change in post exercise values may not be seen with heel raises compared to treadmill exercise in a healthy population where exercise capacity may differ.

Additional variables such as exercise tolerance, sedentary lifestyle, and body mass index were also not measured before assessment so the influence of these parameters on post exercise TBI and TP is unknown. A validated questionnaire for measuring presence of claudication was not utilized, however a standardized question “do you experience any pain or discomfort in your legs when walking” was asked by an experienced clinician to determine the presence of claudication. Finally, the heel raise method of exercise may not be suitable for all populations at risk of PAD, including the frail and those at high risk of falls.

## Conclusion

In a community-based population with resting TBI ≥0.60 and ABI between 0.90 to 1.40, thirty seconds of heel raises resulted in a 4.50 mmHg decrease in TP and 0.07 decrease in the TBI value from the resting measurements. Resting TBI was found to predict 22.40% of the variance in post exercise TBI measurement. The change found between resting and post exercise TBI is consistent with changes seen in the TBI following treadmill walking for up to 5 min and the 6 min walk test. Thirty seconds of heel raises may represent a time efficient alternative method of exercise, when performing post exercise TP and TBI measurements.
